# Supplementary material for: Mpf1 affects the dual distribution of tail-anchored proteins between mitochondria and peroxisomes
Source: EMBO Rep. 2025 Apr 2;26(10):2622–53. doi: 10.1038/s44319-025-00440-6 (PMC12116889; doi:10.1038/s44319-025-00440-6)
Supplement: Supplementary file 3 — Expanded View Figures [file 44319_2025_440_MOESM3_ESM.pdf]

Expanded View Figures

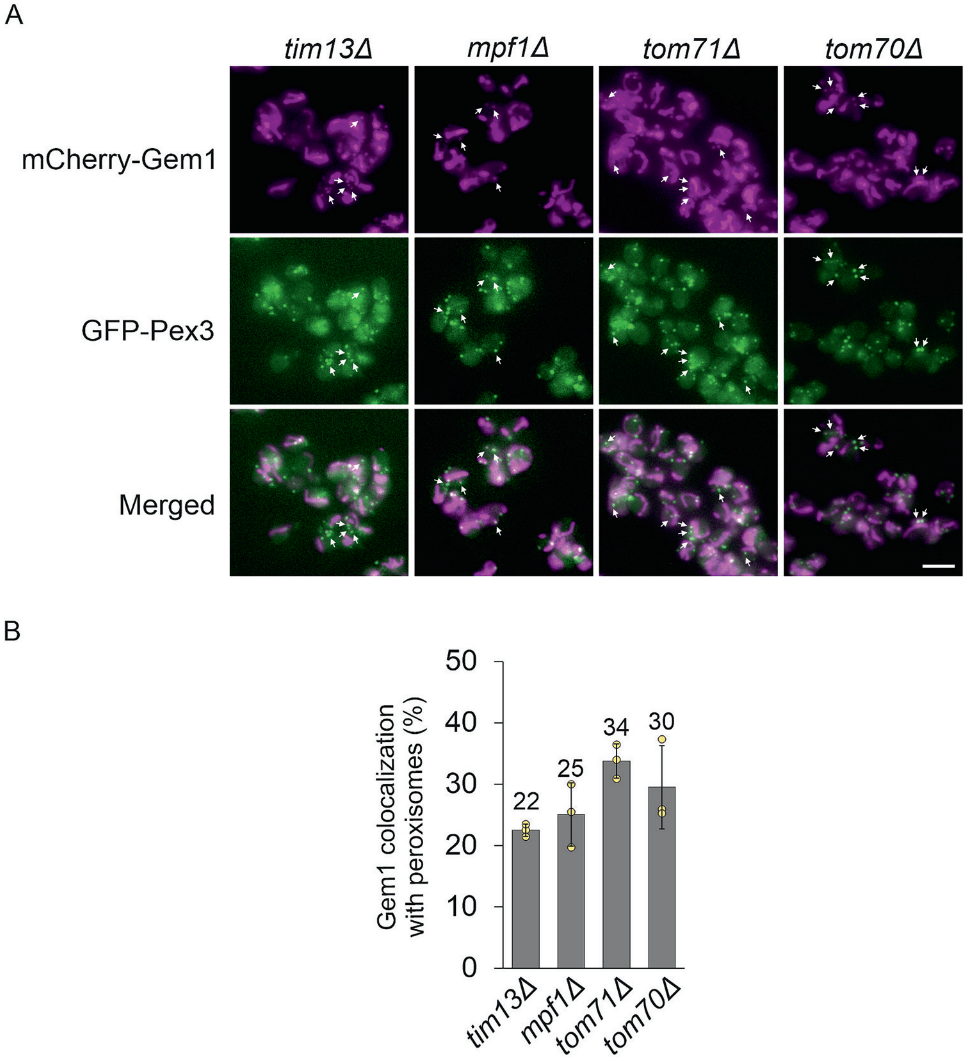

Figure EV1. The hits influencing Fis1's distribution to mitochondria and peroxisomes impact also the dual distribution of another TA protein, Gem1.

(**A**) Representative images of three strains (mpf1Δ, tom71Δ, and tom70Δ) with altered distribution of Gem1 between mitochondria and peroxisomes. The strains co-express mCherry-Gem1 and GFP-Pex3 (as peroxisomal marker). These strains maintain normal mitochondrial morphology and peroxisomes number. tim13Δ cells were used as control. Co-localization of mCherry-Gem1 (as magenta puncta, shown with white arrows) with Pex3-GFP (as green puncta, shown with white arrows) is indicated. Scale bar, 5 µm. (**B**) Quantification of the co-localization of Gem1 with peroxisomes. The total number of peroxisomes (visualized by Pex3-GFP) were counted in 100 cells in three independent experiments. Subsequently, the percentage of co-localization of mCherry-Gem1 puncta with the peroxisomes was determined. Error bars represent ±SD.

 © The Author(s)

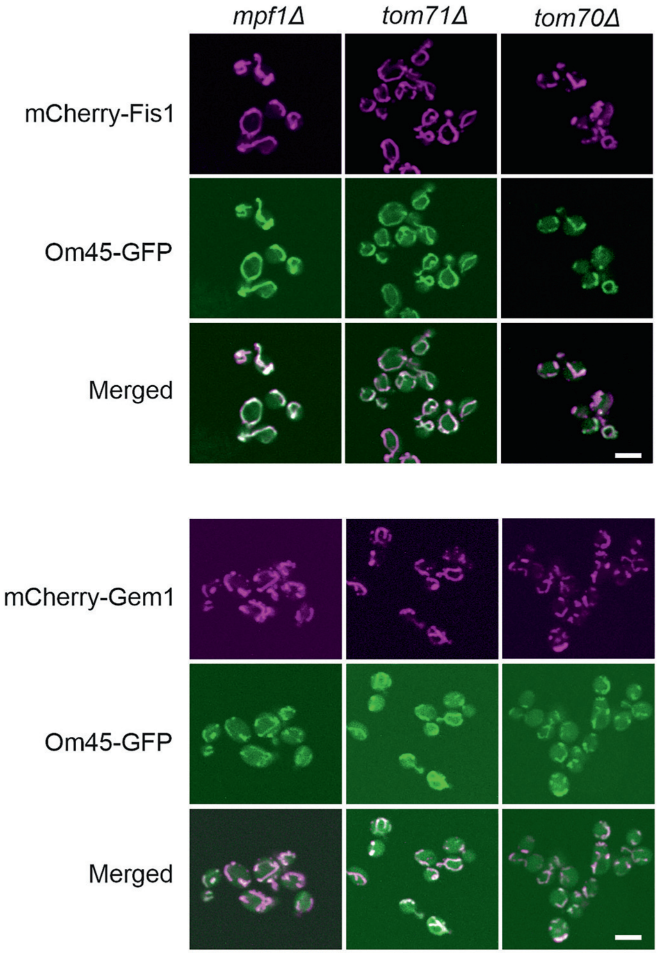

Figure EV2. Regular mitochondrial morphology is observed in *mpf1Δ*, *tom71Δ*, and *tom70Δ* cells.

Mitochondrial morphology was visualized by imaging of mCherry-Fis1 (top panel) and mCherry-Gem1 (bottom panel) in the indicated strains. Om45-GFP served as a marker for mitochondrial structures. Scale bar, 5 µm.

© The Author(s)

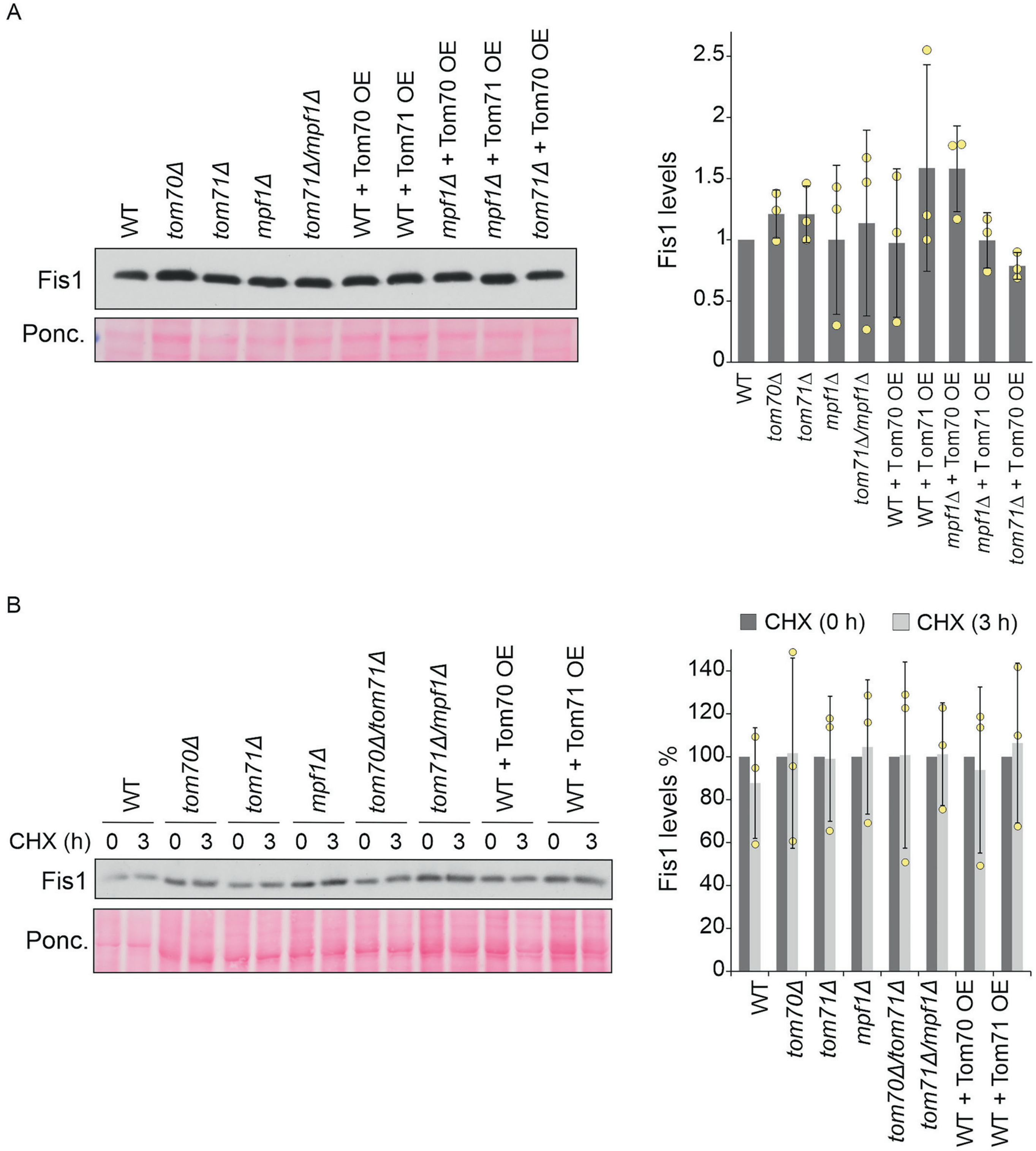
A
WT
tom70Δ
tom71Δ
mpf1Δ
tom71Δ/mpf1Δ
WT + Tom70 OE
WT + Tom71 OE
mpf1Δ + Tom70 OE
mpf1Δ + Tom71 OE
tom71Δ + Tom70 OE
Fis1
Ponc.
Fis1 levels
0
0.5
1
1.5
2
2.5
B
CHX (h)
0 3
Fis1
Ponc.
tom70Δ/tom71Δ
CHX (0 h)
CHX (3 h)
Fis1 levels %
0
20
40
60
80
100
120
140

 © The Author(s)

Figure EV3. The absence or overexpression of Tom70, Tom71, and Mpf1 affect neither the steady-state levels of Fis1 nor its stability.

(**A**) Left panel: Mitochondria isolated from the indicated cells (grown on oleate-containing medium) were analyzed by SDS-PAGE and immunodecoration with anti-Fis1 antibody. The Ponceau stain is shown as loading control. Right panel: the band corresponding to Fis1 in three independent experiments as the one shown in the left panel were quantified and corrected for loading variations according to the Ponceau stain. The intensity of the band in wild type cells was set as 1. Error bars represent ±SD. (**B**) Left panel: The indicated cells were grown on glucose and then at time = 0 the translation inhibitor cycloheximide (CHX) was added and cells were further incubated for three hours. Then, cellular proteins were extracted by alkaline lysis and analyzed by SDS-PAGE and immunodecoration with an antibody against Fis1. The Ponceau stain is shown as loading control. Right panel: the bands corresponding to Fis1 in three independent experiments as the one shown in the left panel were quantified and corrected for loading variations according to the Ponceau stain. For each strain, the intensity of the Fis1 band at time = 0 was set as 100%. Error bars represent ±SD.

© The Author(s)

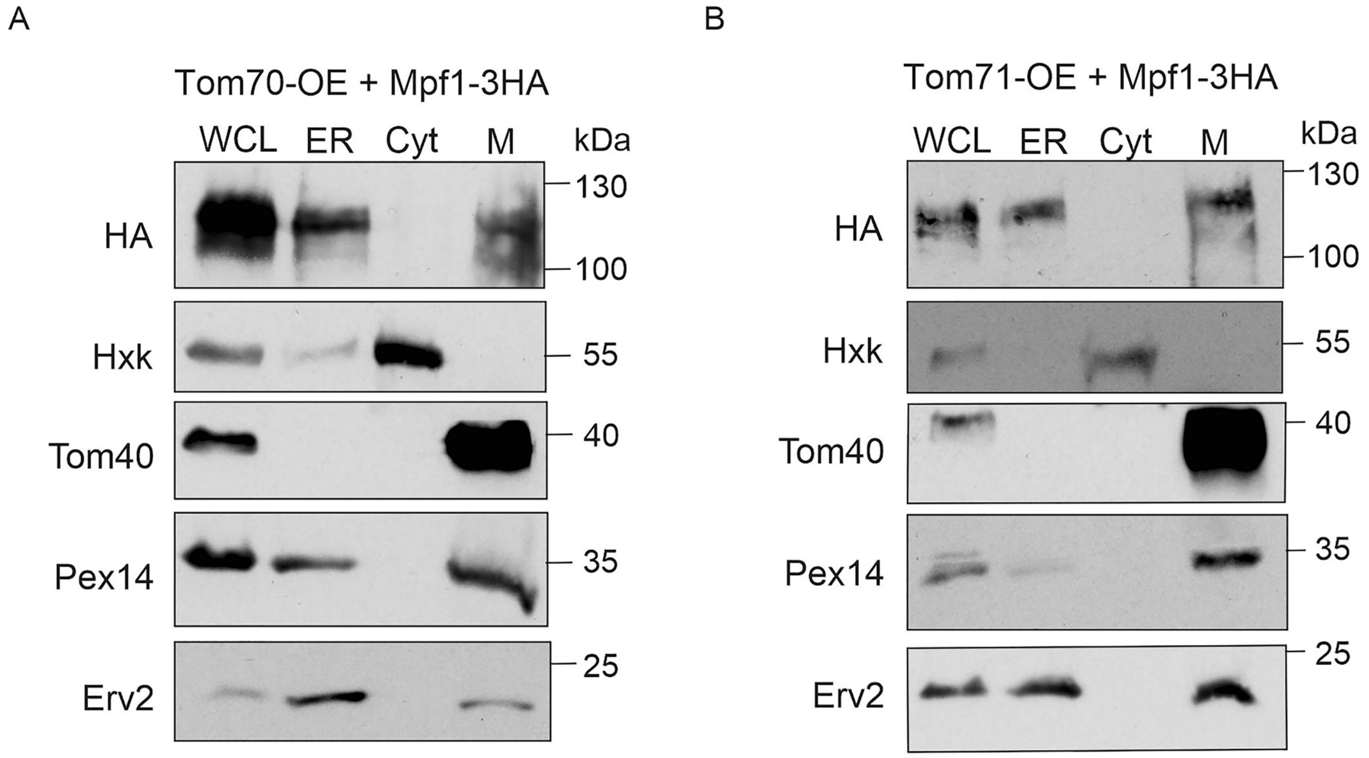

Figure EV4. Overexpression of either Tom70 or Tom71 does not affect the subcellular location of Mpf1.

Cells co-overexpressing Mpf1-3HA with either Tom70 (**A**) or Tom71 (**B**) were subjected to subcellular fractionation. The isolated fractions of whole cell lysate (WCL), microsomes (ER), cytosol (Cyt), and mitochondria (M) were analyzed by SDS-PAGE and immunodecoration with the indicated antibodies. Hexokinase (cytosol), Tom40 (mitochondria), Pex14 (peroxisomes), and Erv2 (ER) were used as marker proteins.

 © The Author(s)

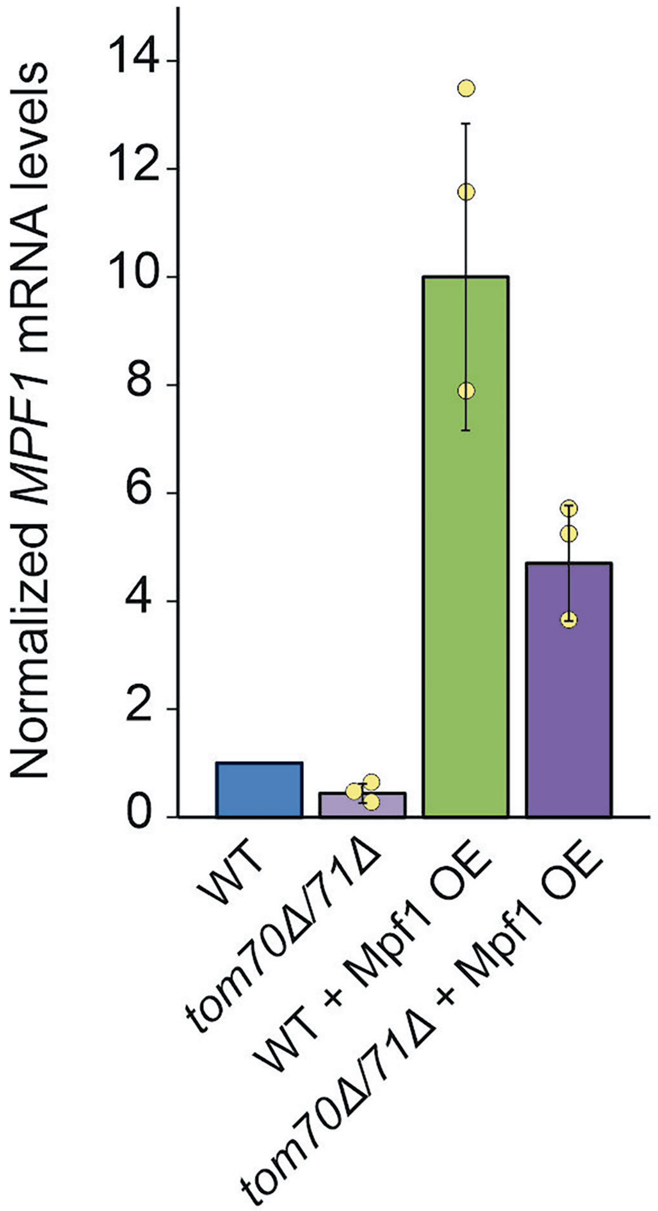

Figure EV5. The transcript levels of Mpf1 are reduced in *tom70/71Δ* cells.

RT-qPCR analysis was performed in both WT and *tom70/71Δ* cells to detect transcript levels of either endogenous MPF1 or upon transformation of cells with overexpression plasmid encoding MPF1-3HA. The transcript levels of ACT1 (encoding the abundant protein Actin) served as a reference. The results of three independent experiments are depicted. Error bars represent ±SD.

© The Author(s)

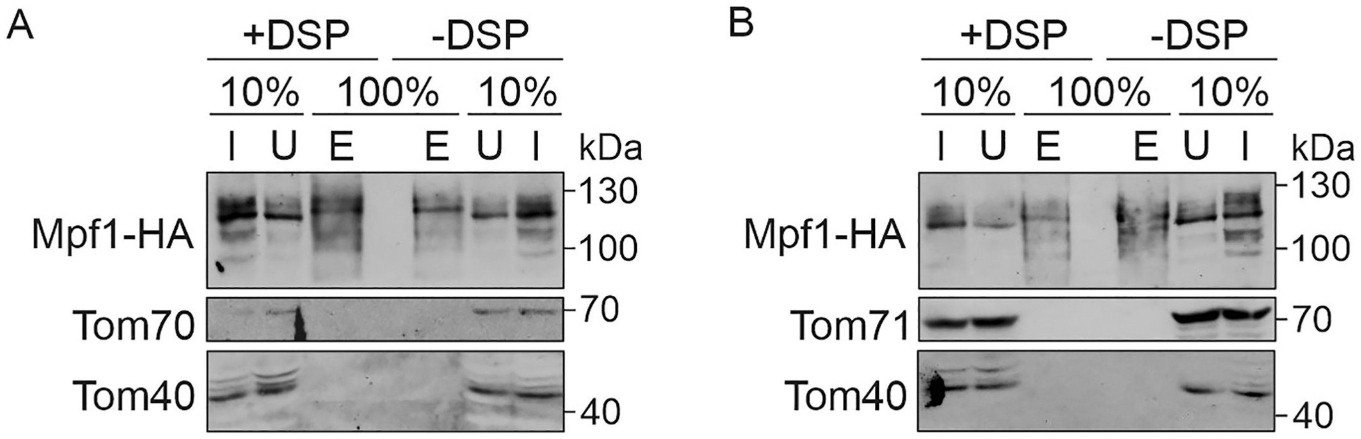

Figure EV6. Absence of detected interaction of Mpf1 to either Tom70 or Tom71.

(**A**, **B**) WT cells co-expressing Mpf1-HA and either Tom70-Flag (**A**) or Tom71-Flag (**B**) were lysed with 1% Triton X-100 and incubated with HA-beads in the absence (−DSP) or presence of the chemical crosslinker DSP (+DSP). Fractions representing the input (I, 10%), unbound material (U, 10%), and the eluate (E, 100%) were analyzed by SDS-PAGE and immunodecoration with the indicated antibodies.

 © The Author(s)
